# Biosorption of nickel, cobalt, zinc and copper ions by *Serratia marcescens* strain 16 in mono and multimetallic systems

**DOI:** 10.1007/s10532-021-09964-9

**Published:** 2021-10-17

**Authors:** A. Díaz, J. Marrero, G. Cabrera, O. Coto, J. M. Gómez

**Affiliations:** 1 Metal Biotechnology Laboratory, Faculty of Biology, University of Havana (Cuba), 25th Street #455 Vedado, 10400 La Habana, Cuba; 2 Biological and Enzymatic Reactors Group, Department of Chemical Engineering and Food Technology, Faculty of Sciences, Puerto Real, 11510 Cádiz, Spain

**Keywords:** Biosorption, Heavy metals, *Serratia marcescens*, Waste liquor (WL), Multimetallic systems

## Abstract

The metallurgical industry is one of the main sources of heavy metal pollution, which represents a severe threat to life. Metals can be removed from aqueous solutions by using microbial biomasses. This paper analyses the heavy metal biosorption capacity of *Serratia marcescens* strain 16 in single and multimetallic systems. The results obtained show that Co(II), Ni(II) and Zn(II) biosorption in monometallic systems is two to three times higher than in the presence of bi-metallic and multimetallic solutions. Fourier transform infrared spectroscopy confirmed that carbonyl, carboxyl and hydroxyl were the main functional groups, as well as the amide bands I and II involved in metal uptake, which are present in external structures of the bacterial cell. The results obtained demonstrated the viability of *S. marcescens* strain 16 as a biosorbent for the design of eco-friendly technologies for the treatment of waste liquor.

## Introduction

Heavy metals are considered to be among the most hazardous pollutants and represent a severe threat to life and ecological balance. The deleterious effects of heavy metals on biological systems are complex and usually dependent on their chemical form, solubility and concentration, which determine their availability to organisms (Gikas 2008).

One of the modern trends in the mining and metallurgical industries is the reduction of waste production because of its negative impact on the environment. Cuba owns one of the largest lateritic Ni(II) and Co(II) deposits in the world (Kuck 2013; Shedd 2013) located in the region of Moa. Industrial processing of the lateritic minerals through acid pressure technology generates considerable amounts of a liquid waste known as waste liquor (WL). This WL contains toxic metals such as Ni(II) (25–28 mg L^−1^), Co(II) (0.2–5.6 mg L^−1^), Zn(II) (15–19 mg L^−1^) and Cu(II) (3–9 mg L^−1^) (Sosa 2006; Sosa and Garrido 2009). Different studies have been carried out to find alternatives for the treatment of this waste. Chemical procedures applied by Sosa et al. (Sosa 2006; Sosa and Garrido 2009) showed good metal recovery from the WL for Cu(II) (81.2%), Zn(II) (75%), Co(II) (79–82%) and Ni(II) (82–84%). However, these methods still pose a threat to the environment, as well as increasing the cost of the procedure, due to the use of chemical agents and specific temperature and shaking conditions for selective chemical precipitation. For this reason, there is still need for eco-friendly and cost-effective technologies that could help to minimize the harmful impact of this residue. Nowadays, the application of nanoparticles in heavy metal adsorption is of contemporary interest (Singh et al. 2021a; Pereira de Sá Costa et al. 2021; Singh et al. 2021b).

At the same time, natural biosorbents such as *Azadirachta indica* twig ash (Singh and Mishra 2020a) or activated carbon derived from *Tectona grandis* sawdust (Singh and Mishra 2020b) are promising alternatives to be used in the simultaneous removal of heavy metal ions.

A feasible alternative could be biosorption using bacterial strains. Among the main advantages of this technology are its low cost, short operation time, high metal binding efficiency, improved selectivity for specific metals of interest, the reusability of biosorbents and the fact that it does not produce secondary toxic compounds (Volesky 2007). Additionally, it is particularly useful to treat diluted liquid wastes with very low metal concentrations (< 100 mg L^−1^) (Kratochvil and Volesky 1998).

Moreover, the study of heavy metal biosorption shows promising results when using multimetallic systems since most heavy metal polluted effluents carry more than one of these compounds in solution (Pereira de Sá Costa et al. 2021), like the WL from Moa (Cuba). In these systems, biosorption of the metal of interest depends not only on the biomass surface properties and physicochemical parameters of the solution, but also on the number of metal cations present and their concentrations which determine whether biosorption will become competitive with one solute competing with another to occupy the binding sites (Vijayaraghavan and Yun 2008).

An increasing number of research studies have been conducted to study heavy metal biosorption using bacteria (Gialamouidis et al. 2009; Marrero et al. 2009; Narasimhulu and Setty 2012; Singh and Gadi 2012), fungi (Chen and Wang 2007; Cayllahua and Torem 2010; Pundir and Dastidar 2010), algae (Vijayaraghavan et al. 2005; Lezcano et al. 2011; Santos et al. 2012) and some agricultural wastes as biosorbents. Bacteria offer certain advantages due to their small size, adaptability and growth conditions which makes them relatively easy to obtain as biosorbents (Urrutia 1997; Wang and Chen 2009). They can be highly effective in the uptake of soluble and particulate forms of metals. Different species of *Bacillus, Pseudomonas, Streptomyces, Escherichia, Micrococcus*, among others, have been reported as good biosorbents for a variety of heavy metals (Kratochvil and Volesky 1998; Wang and Chen 2009).

*Serratia marcescens* is a Gram-negative rod-shaped bacterium of the *Enterobacteriaceae* family that has been isolated from different environmental and nosocomial sources. Its importance as an opportunistic human pathogen has been acknowledged in the last decades, when *S. marcescens* has been signalled as responsible for a range of symptoms in hospitalized patients. However, some of these virulence factors, most notably those of secondary importance, may also confer competence for any strain to strive in different hosts like insects and plant surfaces, and also soil and water (Abreo and Altier 2019).

Being a ubiquitous microorganism, environmental *S. marcescens* has been isolated from water and soil, plants, insects, food and machinery. Plant roots and their adjacent soil—the rhizosphere—can host *S. marcescens* and other species that positively interact with plants, enhancing nutrition, stress tolerance and health and therefore have been considered plant growth promoting rhizobacteria (PGPR).

Therefore, taking advantage of these natural properties that it possesses and the genetic studies carried out, it has been shown to be highly resistant to certain heavy metals (Zakeri et al. 2010).

*Serratia* representatives have been proposed as good biosorbents for radionuclides (Macaskie et al. 2010; Yoonaiwong et al. 2011). *S. marcescens* has been reported as a chromium and molybdenum reducing bacterium (Campos et al. 2005; Shukor et al. 2009) as well as a bioindicator of Pb(II), Cr (VI) and Cd(II) (Cristani et al. 2011).

*Serratia marcescens* strains 16, C-1 and C4 isolated from a nickel deposit in Moa (Cuba) were classified as highly Ni(II) and Co(II) resistant bacteria and showed removal capacity of nickel and cobalt ions from monometallic systems in the presence of 50 mg L^−1^ as an initial concentration (Marrero et al. 2009). The presence of the genetic determinant *ncr*ABC in *S*. *marcescens* strains 16, C-1 and C4 was related to the removal capacity of nickel and cobalt that could be of interest in environment bioprocesses (Marrero et al. 2009).

Some research work in the literature deals with the possible use of metal resistant bacteria as potentially efficient biosorbents due to the presence of specific heavy metal binding sites on the cell surface (Kao et al. 2008). *Escherichia coli strain* WS11 isolated from agricultural soil irrigated with industrial wastewater showed significant heavy metal resistance and its Ni(II) and Cd(II) biosorption capacity was increased from 6.96 to 55.31 mg g^−1^ of cells and 4.96 to 45.37 mg g^−1^ of cells, respectively, at a concentration ranging from 50 to 400 µg ml^−1^ after 2 h of incubation between biosorbent and sorbate in single and bi-metallic systems (Ansari and Malik 2007). Additionally, recombinant *E. coli* strains over-expressing metal-binding (MerP) protein, showed a marked improvement in the adsorption capacity of Zn(II) and Cr (III) when compared to the control host strain (Kao et al. 2008). *Enterobacter cloacae* strain P2B, a lead resistant bacterium, showed a capacity to sequester a higher amount of this heavy metal (17% lead by weight) than the non-resistant strain of *E. cloacae* due to the production of lead-enhanced exopolysaccharide (Naik et al. 2012). Some studies on biosorption capacities were carried out by Kaduková and Horváthová (2013), in which they studied the biosorption of copper, zinc and nickel from multi-ion solutions by a microscopic green algae *Chlorella kessleri.*

The aim of this work was to determine Ni(II), Co(II), Cu(II) and Zn(II) biosorption capacity by *S. marcescens* strain 16 in single and multimetallic systems, and to evaluate its potential application in biosorption technology design.

## Materials and Methods

### Biosorbent preparation

*Serratia marcescens* strain 16 belongs to the microbial collection of the Biology Faculty, University of Havana, and was isolated from serpentine deposits located in Moa (Cuba) (Marrero et al. 2009).

Cultures were prepared in a liquid medium and incubated with shaking (150 rpm) at different time intervals at 37 °C. The cells were harvested when they reached an OD value close to 0.6, by centrifugation at 10,000 rpm, 10 min and 4 °C. Subsequently, they were washed with sterile distilled water and dried with dry heat in an oven at 60 °C. The dry biomass was manually crushed and stored in a desiccator until its use.

### Batch biosorption experiments

Biosorption experiments were conducted using a previously defined biomass concentration of 0.6 g L^−1^, a temperature of 30 °C, pH of 4.5, contact time of 2 h and shaking at 120 rpm in a rotary shaker (Diaz 2013). After contact, the biomass was harvested by centrifugation at 8,000 rpm for 10 min to determine the heavy metal concentrations remaining. Biosorption capacity (q) was determined as previously established (Volesky 2007) according to Eq. (1):

1 $$q = \frac{{V \times (C_{{\text{i}}} - C_{{\text{f}}} )}}{{X_{0} }}$$

where q is the biosorption capacity expressed as mg of metal adsorbed per gram of biomass; V is the volume; *C*_i_ and *C*_f_ are the initial and final metal concentrations, respectively and X_0_ is the initial biomass concentration. Mean values of q obtained for every metal were compared using analysis of variance (ANOVA) (p < 0.05). The percent of metal removal was calculated as indicated in Eq. (2):

2 $${\text{RE}}\;(\%) = \frac{{(C_{{\text{i}}} - C_{{\text{f}}} ) \times 100}}{{C_{{\text{i}}} }}$$

Heavy metal solutions were prepared using analytical grade salts of CoSO_4_, NiSO_4_, CuSO_4_·0.5H_2_O and ZnSO_4_. Metal concentrations employed were selected according to the chemical composition of the liquor WL (2 mg L^−1^ of Co(II), 25 mg L^−1^ of Ni(II), 15 mg L^−1^ of Zn(II) and 9 mg L^−1^ of Cu(II) (Sosa 2006). These metal concentration values were also employed to prepare the bi- and multimetallic dissolutions. Specifically, bi-metallic solutions were used [Ni(II) (25 ppm) + Co(II) (2 ppm)] and multimetallic [Ni(II) (25 ppm) + Co(II) (2 ppm) + Cu(II) (9 ppm) + Zn(II) (15 ppm)].

### Fourier transform infrared spectroscopy (FTIR)

Infrared absorption spectra were acquired with a FTIR-8400S Spectrometer (Shimadzu Corporation, Kyoto, Japan). The data acquisition was carried out through the transmittance mode and air was used for background substraction. The *S. marcescens* biomass without any adsorbed metals prepared as previously described was used as a negative control. The spectra were collected after 20 scans of 1 s at 4 cm^−1^.

### Scale up of the biosorption process

**Fig. 1:**
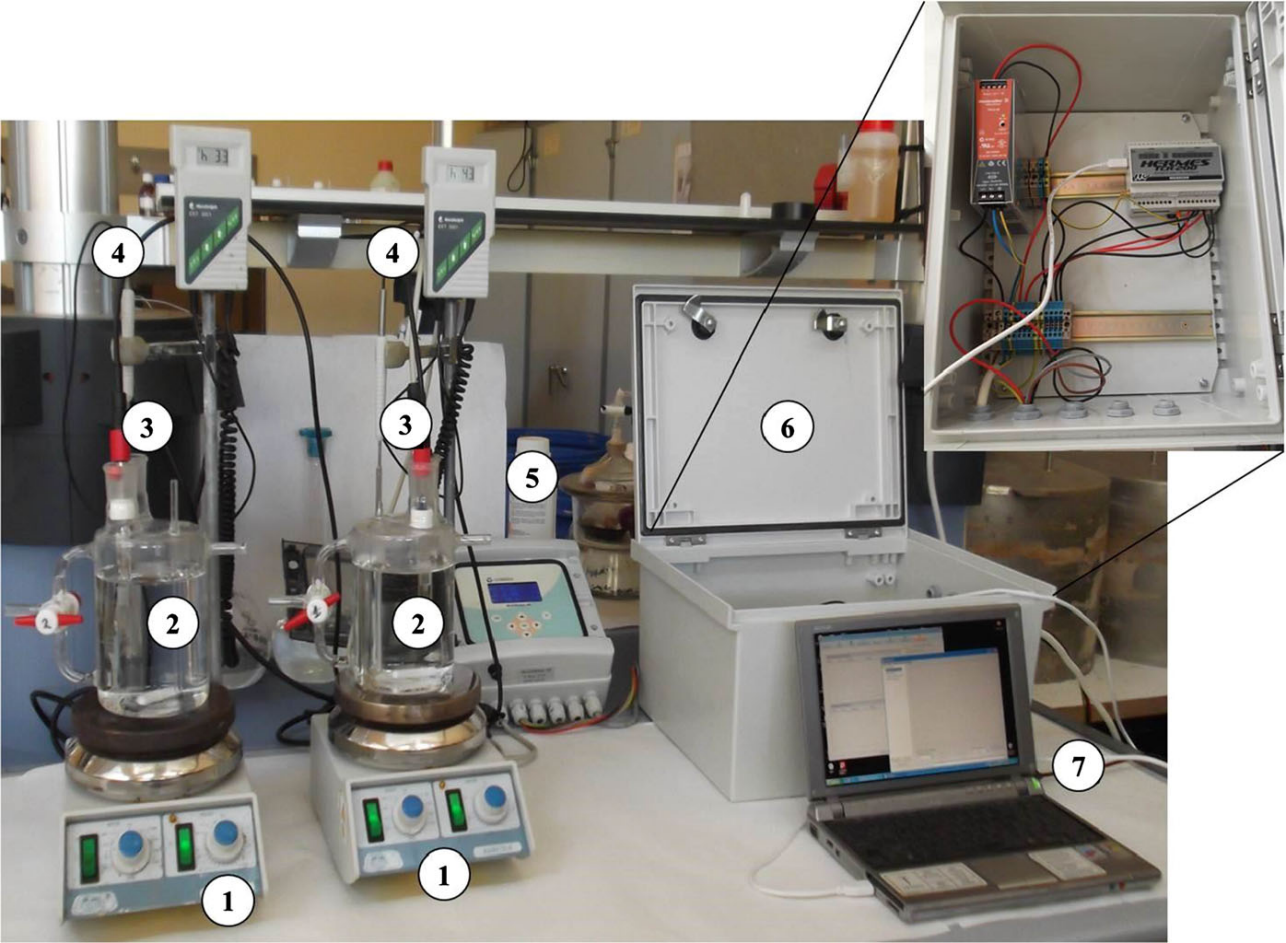
Experimental assembly of 1 L bioreactors. 1, 4: temperature control system, 2: 1 L reactors; 3, 5: pH electrodes coupled to a multimeter, 6, 7: data loader and storing system

Scale up of the process was carried out in 1 L bioreactors with an effective volume of 750 mL using single and multimetallic systems. Glass reactors containing the metal solutions were placed on heating plates to keep the temperature constant at 30 °C. pH electrodes and temperature control were used and coupled to a multimeter for pH and temperature monitoring (Fig. 1). All the information obtained was stored in a data collection system for further analysis. Samples were taken at different times of contact between biomass and sorbate (15 min, 30 min, 1 h and 2 h). Bacterial biomass was separated by centrifugation and the heavy metal concentrations remaining in the supernatant were determined by ICP-OES (Inductively Coupled Plasma Optical Emission Spectrometer).

## Results and discussion

### Biosorption of heavy metals in mono, bi and multimetallic solutions

**Fig. 2:**
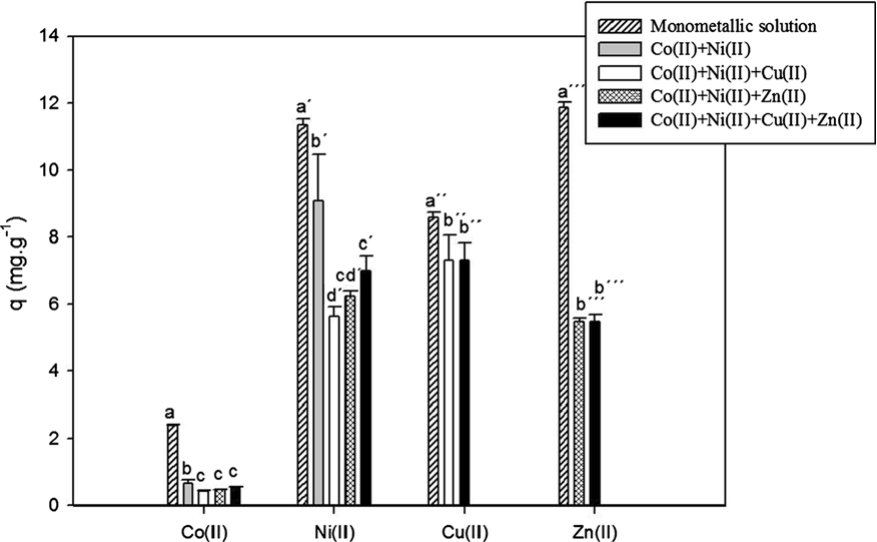
Biosorption capacity of the metal ions by *S. marcescens* strain 16 biomass in single and multimetallic systems: initial concentrations 2 mg L^−1^ Co(II), 25 mg L^−1^ Ni(II), 9 mg L^−1^ Cu(II) and 15 mg L^−1^ Zn(II), contact time 2 h, biomass 0.6 g L^−1^. Bars represent the average of three determinations ± SD. Different letters correspond to statistically significant differences

Figure 2 shows the metal removal capacity of a *S. marcescens* strain 16 biomass in the presence of single, bi and multimetallic solutions. The highest values of q were obtained in mono metal solutions (2.3 mg g^−1^ Co(II), 11.4 mg g^−1^ Ni(II), 8.6 mg g^−1^ Cu(II) and 11.9 mg g^−1^ Zn(II). The comparison between metal biosorption performances of a biosorbent in the presence of different sorbates should be made in similar conditions, including its initial concentration (Volesky 2007). This study did not compare different sorbates since only one WL dissolution was designed, presenting variable concentrations of metal ions. Therefore, it was expected that biosorption capacity behaviour would be qNi > qZn > qCu > qCo, since the concentration values used were [Ni(II)] > [Zn(II)] > [Cu(II)] > [Co(II)]. However, it was noted that even when the concentration of Zn(II) ions (15 mg L^−1^) was lower than the Ni(II) concentration used (25 mg L^−1^), biosorption capacity for these ions showed very similar values with monometallic solutions (Fig. 2). This behaviour suggests a possible higher affinity of the biosorbent for Zn(II) ions under the experimental conditions used.

Biosorption capacity for Co(II), Ni(II) and Zn(II) in monometallic systems proved to be two to three times higher than those values obtained for these metal cations in bi- and multimetallic solutions, possibly due to competition between these metals for binding sites on the biosorbent. This fact has been confirmed in other studies using another bacterial biomass as a biosorbent. For instance, Narasimhulu and Setty (2012) determined Cr (VI) and Cd(II) biosorption by a dead biomass of *Pseudomonas fluorescens* and *Pseudomonas putida* respectively, which turned out to be remarkably lower when combined with other heavy metals like Cu(II) and Ni(II). *Brevundimonas vesicularis* showed a lower Ni(II) and Cu(II) biosorption capacity in the presence of both metals than in single metal solutions (Singh and Gadi 2012).

It has been documented in the literature that effective binding sites available for a single metal can be reduced when other metal cations are present, due to the competition phenomena. This is influenced by each metal concentration, as well as by the specific characteristics of metal cations such as ionic radius and covalent index (directly dependent of electronegativity) (Chen and Wang 2007). The metals used in the present study were all divalent cations and have very similar ionic radii, however, biosorption capacity for these ions is correlated to their covalent indices and decreases in the following order: Cu(II) > Zn(II) > Co(II) > Ni(II) (Chen and Wang 2007). Small differences are observed between the values of biosorption capacity (q) for Cu(II) ions using single or multimetallic solutions (Fig. 2). This could be related to the specific binding properties of the biomass for this particular ion, which do not decrease in the presence of other competing metal cations (Chen and Wang 2007), and are relative to the covalent index of this ion compared to the others used in the present study.

### Characterization of the biomass

**Fig. 3:**
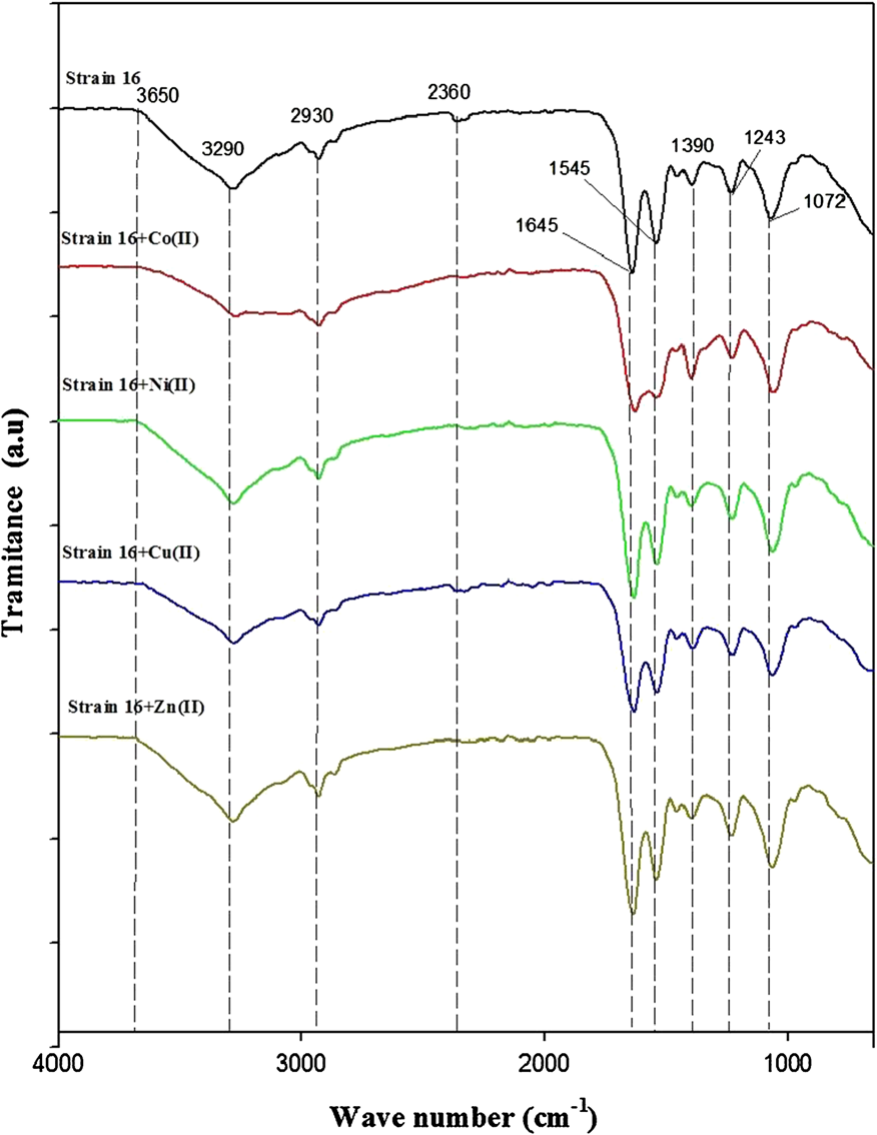
FTIR spectra for heavy metal loaded biomass [Strain 16 + Co(II); Strain 16 + Ni(II); Strain 16 + Cu(II) and Strain 16 + Zn(II)] in contrast with negative control (Strain 16)

The FTIR spectra of unloaded and metal loaded *S. marcescens* biomasses in the range of 500–4000 cm^−1^ were recorded to check the involvement of functional groups that are usually responsible for the biosorption process occurring in different structures of the bacterial cell envelope. Figure 3 presents the spectra obtained for a metal free biomass (negative control) in contrast to a metal loaded biomass for each metal cation studied. Table 1 shows the characteristic bond and functional groups corresponding to each band found in the *S. marcescens* strain 16 biomass spectra.

**Table 1:** FTIR vibrational bands obtained for metal loaded biomass in contrast with negative control (strain 16)

Wave number (cm^–1^)					Corresponding functional group
Strain 16	16 + Co(II)	16 + Ni(II)	16 + Cu(II)	16 + Zn(II)	
1072	1048	1054	1054	1054	–C–N, –C–O
1243	1220	1220	1220	1220	–P=O
1390	1390	1390	1390	1390	–C–H (–CH_2_, –CH_3_)
1545	1527	1533	1533	1533	Amide band II
1645	1622	1633	1633	1633	–C=O (COO^–^), Amide band I
2360	–	–	2360	–	–C≡N
2930	2930	2930	2930	2930	–C–H (–CH_2_, –CH_3_)
3290–3650	3290–3650	3290–3650	3290–3650	3290–3650	–NH; –OH^–^

Figure 3 shows certain differences between the biomasses obtained after contact with each metal solution and the negative control (without metal ions) through the stretching and/or displacement of the bands. As expected, there is evidence of interactions between metal cations and the different functional groups present in the biomolecules of *S. marcescens’* outer structures: Gram negative cell wall and membrane cell. Among the vibrational bands obtained are those corresponding to hydroxyl, carbonyl, carboxyl, amide, imidazole, phosphate and phosphodiester groups, which have been established in different studies in the literature as the main functional groups responsible for a biosorption process with microorganisms (Volesky 2007; Cayllahua and Torem 2010; Lee et al. 2011; Sabat et al. 2012; Santos et al. 2012).

The higher differences between the metal loaded biomass and the negative control were obtained after contact with Co(II) ion solution. The vibrational bands corresponding to the carbonyl, carboxyl and hydroxyl groups, as well as the amide I and II bands, present main differences with the negative control and even with the spectra obtained for Ni(II), Cu(II) and Zn(II) loaded biomasses. This could suggest a possible differential interaction between Co(II) ions and the biosorbent through the involvement of these functional groups.

### Desorption of Ni(II) and Co(II)

**Fig. 4:**
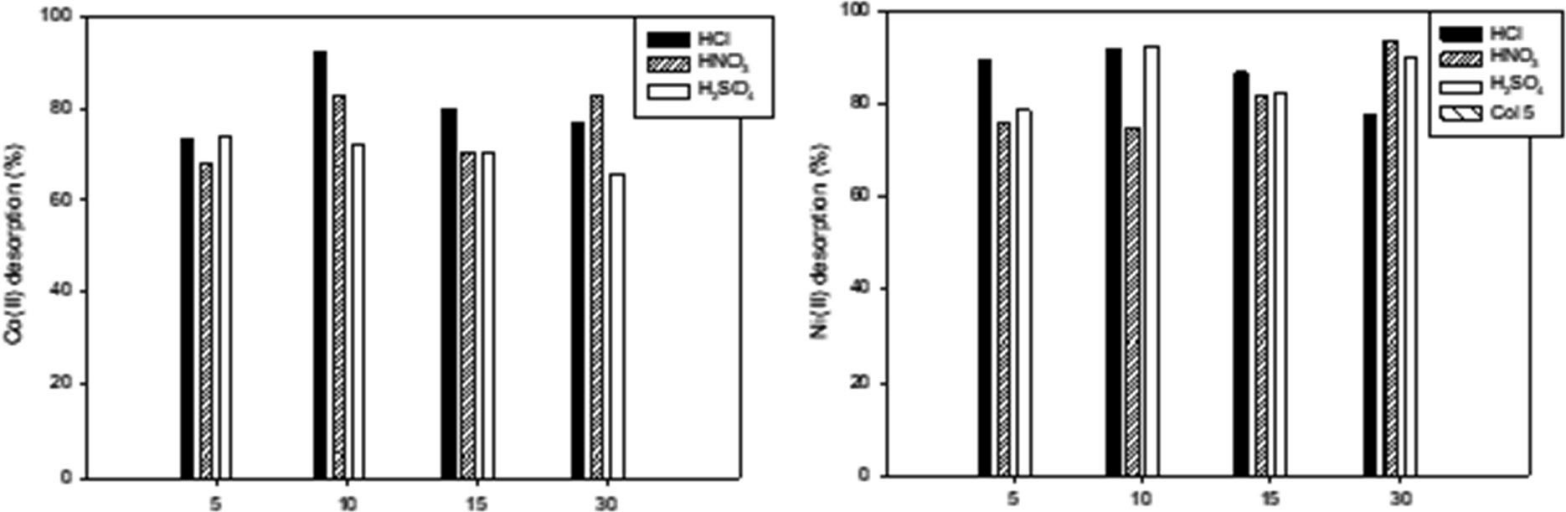
Co(II) and Ni(II) desorption using 0.1 mol L^−1^ HCl, HNO_3_ and H_2_SO_4_ solutions at different contact times, using 1 ml of eluent agent incubated at 30 °C, 120 rpm

Figure 4 shows the desorption (%) of Ni(II) and Co(II) ions using 0.1 mol L^−1^ solutions of H_2_SO_4_, HNO_3_ and HCl. Desorption tests showed that HCl was the best eluent for the two metals tested, more than 70% of Co(II) ions and 85% of Ni(II) ions was achieved within the first 5 min of contact between metal loaded biomass and 0.1 mol L^−1^ HCl solution. More than 90% of both metals were recovered at 10 min with a higher desorption efficiency than HNO_3_ and H_2_SO_4_ solutions (Fig. 4). There was no increase in desorption efficiency at higher contact times. The results concur with those reported for the recovery of Cd(II), Cu(II), Pb(II), Ni(II), Co(II) and Zn(II) adsorbed by different microbial biosorbents (Pundir and Dastidar 2010; Karaoglu et al. 2011; Lezcano et al. 2011; Yoonaiwong et al. 2011). These authors also employed HCl solutions at different concentrations as eluent agents and achieved high efficiency of desorption (greater than 80% in all cases).

Desorption of the adsorbed metals from biosorbents is one of the most attractive issues of heavy metal biosorption strategies based on microbial biomass. It is economically favourable to obtain low volumes of highly concentrated wastes from which heavy metal recovery can be achieved by a combination of further chemical or physical methods. However, another widely discussed aspect, is the possibility of microbial biosorbent regeneration after desorption treatment. In this present study, there was no possibility to reuse the *S. marcescens* biomass in any further biosorption cycle due to the loss of biosorptive capacity after treatment with HCl solution (data not shown). Chergui et al. (2007) who found a decrease of over 50% in Cu(II) and Zn(II) biosorption capacity of *Streptomyces rimosus* biomass after treatment with HCl solution made similar observations. The treatment of the biosorbent with an inorganic acid could provoke irreversible damage due to structural changes of the active centres, or cause blockage of those sites due to the inefficiency of the eluent, leaving fewer active sites available for a new sorption cycle (Lin and Lin 2005; Vijayaraghavan et al. 2005). Similar behaviour to that observed in the present study was evidenced for Cu(II) recovery from *Pseudomonas* sp. and *Staphylococcus xylosus* biomass using HNO_3_ as an eluent agent (Gialamouidis et al. 2009). Analogous results have been reported for biosorbents from different origins, for example, Lezcano et al. (2011) reported a loss of biosorptive properties of the combined algal plant biosorbent used for the removal of Cd(II), Cu(II) and Pb(II), after recovery of adsorbed metals with HCl. Future studies might be conducted in this area, in order to achieve desorption of the metal from the biosorbent without losing biosorption efficiency.

### Scale up of the biosorption process using mono and multimetallic solutions

In general, biosorption studies at reactor scale have been conducted in two ways, the so-called column system and the batch system. Most studies use batch cultures to evaluate different parameters of the biosorption process and optimize conditions while column design is usually preferred for scale-up testing. However, in the present study, batch conditions were selected for the preliminary study at a bioreactor scale, and the optimal parameters for biosorption performance of *S. marcescens* strain 16 were defined at a 10 mL scale, using a batch regime.

**Fig. 5:**
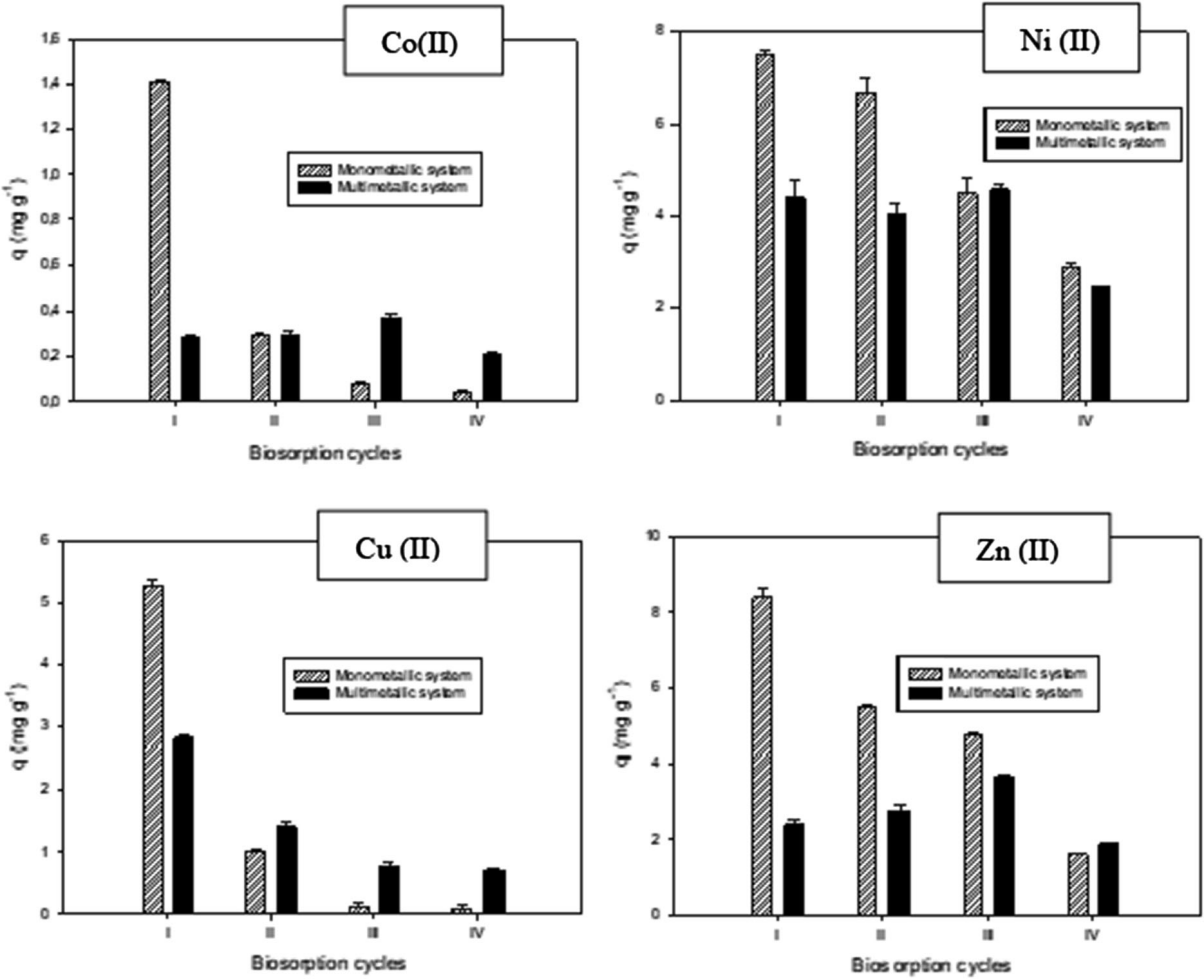
Biosorption capacity for the metal cations studied in mono- and multimetallic solutions through the four biosorption cycles at bioreactor scale. Bars represent medium values of three determinations ± SD

Figure 5 shows biosorption capacity values for every metal cation studied in single or multimetallic systems. Metal biosorption in the presence of monometallic solutions turned out to be higher than in multimetallic solutions in the first biosorption cycle, which agrees with results obtained in flasks under batch operation with 10 mL of effective volume. This fact can be explained considering that in multimetallic solutions competition is established between different metal cations for binding sites on the biomass. In the next three cycles, biosorption capacity behaviour was different for every metal cation studied, depending on the amount of metal adsorbed in the first cycle and how this affects metal concentrations remaining in monometallic or multimetallic solutions. A decrease in the initial metal concentration determines a smaller value of q, because this parameter is directly dependent on the initial metal concentration.

Therefore, for Co(II) and Cu(II) ions, higher values of q were obtained in the first biosorption cycle using monometallic solutions, and lower values were observed in the next three cycles (Fig. 5) probably influenced by the rapid decrease in the remaining heavy metal concentration that takes place in the first biosorption cycle. In contrast, a different behaviour was observed for monometallic systems using Ni(II) and Zn(II) ions (Fig. 5) where a sequential decrease was observed from the first biosorption cycle to the fourth. A possible explanation is that concentrations of these two ions were higher than those of Co(II) and Cu(II), so the first contact metal-biomass did not decrease drastically their concentrations in the simulated residue.

The behaviour observed for q values through the four biosorption cycles when using multimetallic solutions of each metal cation is also notable. Uptake of Cu(II) ions was higher in the first cycle, and slowly decreased with the next three biosorption cycles. This was the “expected” behaviour since q values are directly dependent on initial metal concentrations. In contrast, there were some differences with Co(II), Ni(II) and Zn(II) ions. For these, q values in the first two biosorption cycles were lower than in the third cycle, where an unexpected increase in biosorption capacity occurred, followed by a new decrease in the fourth cycle. This fact could be explained by considering that the metal cations are in competition with each other and selective cation affinity. Cu(II) biosorption was remarkably diminished in the third biosorption cycle, and in fact, no further decrease was observed by the fourth cycle (Fig. 5). So, it is possible that only under this low Cu(II) concentration, do the rest of the metal cations have access to binding sites on the biomass, which could explain the increase in their q values.

All these results suggest that the biosorption capacity of *S. marcescens* strain 16 for Co(II), Ni(II), Cu(II) and Zn(II) decreases when these metal cations are present in multimetallic systems compared with values obtained for each ion in monometallic solutions. This behaviour is influenced by the competition phenomena that take place in multimetallic systems, due to a differential affinity of the biosorbent for each metal cation present.

**Fig. 6:**
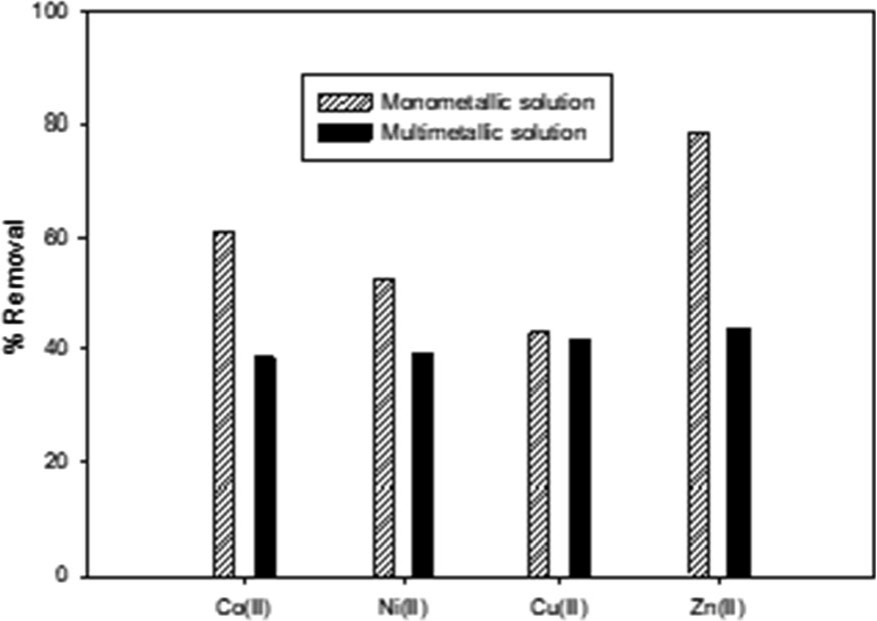
Heavy metal removal at bioreactor scale using single or multimetallic systems. Experiments were conducted in 1 L batch bioreactors, at 30 °C, 120 rpm for 2 h

After four repeated biosorption cycles, it was possible to remove 60.9% of Co(II), 53.6% of Ni(II), 43.1% of Cu(II) and 78.8% of Zn(II) present in monometallic systems, and 39.7% of Co(II), 40.2% of Ni(II), 42.8% of Cu(II) and 44.7% of Zn(II) present in multimetallic systems (Fig. 6). These values are lower than recoveries obtained by using chemical treatment (Sosa 2006; Sosa and Garrido 2009), which exceed 79% for these four metals. Nevertheless, the advantages of biosorption over chemical treatment should also be taken into account. For example, selective chemical precipitation requires 90 g L^−1^ of NaHS as a precipitating agent, as well as a high operating temperature (363 K) and agitation (535 rpm), which increase the cost of the process, especially at an industrial scale. Moreover, the overall procedure generates toxic gases that represent a threat to the environment; so an additional operational cost is the use of gas traps or filters to avoid environmental damage with a new residue. The results obtained in the present study have demonstrated good metal recovery using simple experimental assemblage and no further generation of residue. For these reasons, biosorption using *S. marcescens* strain 16 represents a suitable alternative for waste liquor (WL) treatment, since it would minimize the ecological impact and is economically viable.

The results of this work are the first study at a bioreactor scale of the biosorption process using a *S. marcescens* biomass. The viability of the use of *S. marcescens* strain 16 biomasses in the design of eco-friendly technologies for the environmental remediation of heavy metal contaminated sites has been demonstrated, especially considering the preliminary results obtained in the present study with heavy metal cation mixes.

These facts point towards the possibility of using *S. marcescens* strain 16 for heavy metal removal in multimetallic solutions, as the metal cation concentrations used are very similar to those present in the WL residue. This would help to counteract the negative impact of this waste on the environment, as well as facilitate the recovery of the metal values present. This study is the first report of heavy metal biosorption in multimetallic systems by the *S. marcescens* species.

## Conclusions

In the present study, the biosorption performance of a dead biomass from a native Ni(II) and Co(II) resistant *S. marcescens* strain 16 was studied. The dead biomass of *S. marcescens* strain 16 shows a biosorption capacity for Co(II), Ni(II), Cu(II) and Zn(II) ions present in single or multimetallic systems through interaction with the external functional groups of the cell. Experiments at a bioreactor scale showed that this biosorbent has potential for the design of eco-friendly technologies for environmental remediation of heavy metal contaminated sites. These results are relevant since few studies have been carried out on *Serratia* spp. biosorption performance, and moreover, they include an analysis of the process at a bioreactor scale.

Further research work must study different alternatives for the recovery of metal adsorbed into the biomass of strain 16 without causing any damage to its biosorption performance. New alternatives for pre-treatment and different desorption agents must be evaluated, in order to obtain good metal recovery and reusability of this biosorbent.
